# 14-Year Survey in a Swedish County Reveals a Pronounced Increase in Bloodstream Infections (BSI). Comorbidity - An Independent Risk Factor for Both BSI and Mortality

**DOI:** 10.1371/journal.pone.0166527

**Published:** 2016-11-11

**Authors:** Martin Holmbom, Christian G. Giske, Mats Fredrikson, Åse Östholm Balkhed, Carina Claesson, Lennart E. Nilsson, Mikael Hoffmann, Håkan Hanberger

**Affiliations:** 1 Division of Infectious Diseases, Department of Clinical and Experimental Medicine, Faculty of Medicine and Health Sciences, Linköping University, Linköping, Sweden; 2 Department of Urology and Department of Clinical and Experimental Medicine, Linköping University, Linköping, Sweden; 3 Department of Laboratory Medicine, Karolinska Institute, Stockholm, Sweden; 4 Clinical Microbiology, Karolinska University Hospital, Stockholm, Sweden; 5 Occupational and Environmental Medicine, Department of Clinical and Experimental Medicine, Faculty of Medicine and Health Sciences, Linköping University, Linköping, Sweden; 6 Forum Östergötland, Faculty of Medicine and Health Sciences, Linköping University, Linköping, Sweden; 7 Division of Clinical Microbiology, Department of Clinical and Experimental Medicine, Faculty of Medicine and Health Sciences, Linköping University, Linköping, Sweden; 8 The NEPI foundation, Division of Health Care Analysis, Department of Medical and Health Sciences, Faculty of Medicine and Health Sciences, Linköping university, Linköping, Sweden; Amphia Ziekenhuis, NETHERLANDS

## Abstract

**Objectives:**

we assessed the incidence, risk factors and outcome of BSI over a 14-year period (2000–2013) in a Swedish county.

**Methods:**

retrospective cohort study on culture confirmed BSI among patients in the county of Östergötland, Sweden, with approximately 440,000 inhabitants. A BSI was defined as either community-onset BSI (CO-BSI) or hospital-acquired BSI (HA-BSI).

**Results:**

of a total of 11,480 BSIs, 67% were CO-BSI and 33% HA-BSI. The incidence of BSI increased by 64% from 945 to 1,546 per 100,000 hospital admissions per year during the study period. The most prominent increase, 83% was observed within the CO-BSI cohort whilst HA-BSI increased by 32%. Prescriptions of antibiotics in outpatient care decreased with 24% from 422 to 322 prescriptions dispensed/1,000 inhabitants/year, whereas antibiotics prescribed in hospital increased by 67% (from 424 to 709 DDD per 1,000 days of care). The overall 30-day mortality for HA-BSIs was 17.2%, compared to 10.6% for CO-BSIs, with an average yearly increase per 100,000 hospital admissions of 2 and 5% respectively. The proportion of patients with one or more comorbidities, increased from 20.8 to 55.3%. In multivariate analyses, risk factors for mortality within 30 days were: HA-BSI (2.22); two or more comorbidities (1.89); single comorbidity (1.56); CO-BSI (1.21); male (1.05); and high age (1.04).

**Conclusion:**

this survey revealed an alarming increase in the incidence of BSI over the 14-year study period. Interventions to decrease BSI in general should be considered together with robust antibiotic stewardship programmes to avoid both over- and underuse of antibiotics.

## Background

Bloodstream infection (BSI) is a major cause of morbidity and mortality worldwide and several measures have been taken to prevent BSI and increase survival in sepsis [1–5]. The number of BSIs and number of deaths following BSI in Europe and North America each year have been estimated to be almost 2 million and 250,000 respectively [6]. However, the exact burden of BSI worldwide is difficult to estimate since the incidence of community-onset BSI in low- and middle-income country populations is not known due to lack of population-based BSI studies in these countries [7].

Most studies in high-income countries show continual increases in the incidence of sepsis [8–17]. A decrease in mortality associated with BSI has been reported in some studies from high income countries [18, 19], but increased mortality in others [10].

In this study we assessed the incidence, risk factors and outcome of all BSIs over a 14-year period in a county in Sweden having four hospitals and almost half a million inhabitants. BSIs were defined as either hospital-acquired or community-onset.

## Methods

### Study design and setting

This was a multicentre retrospective cohort study on the incidence and mortality of culture-confirmed bloodstream infections among patients in a Swedish county. The study was carried out in a county with four hospitals in southeast Sweden; a large tertiary care university hospital (608 beds), two secondary care hospitals (310 and 115 beds respectively) and one primary care hospital (14 beds). The number of inhabitants in the county increased from approximately 411,000 to 437,000 over the study period, representing approximately 5% of the Swedish population.

### Data collection

**Fig 1:**
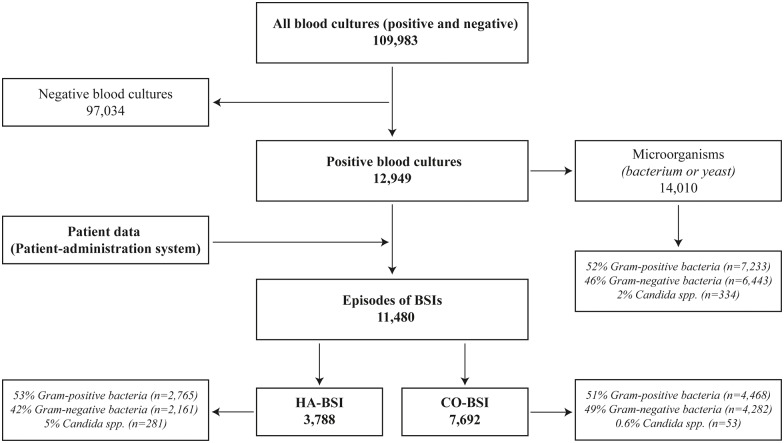
Flow diagram of the study.

From the laboratory database the following data were collected: date of blood culture; number of aerobic and anaerobic blood culture vials taken; site of puncture; species identification; and susceptibility patterns. The dataset was entered into a secondary database where it was linked to the patient-administration system providing the following data for all patients with blood culture taken: gender; age; comorbidity; admitting department; date of admission; date of discharge; and mortality (Fig 1).

### Microbiology

All bacterial isolates were analysed at the species level, and species identification was carried out at the Östergötland Regional Department of Clinical Microbiology, using routine methods.[20]

### Definitions

**Blood culture:** one or more sets of blood cultures taken on the same day. One set comprises one aerobic and one anaerobic blood culture bottle.

**Positive blood culture:** defined as the isolation of microorganisms (one or more bacterial or fungal isolates) from a set of blood cultures taken on the same day.

**Non-significant pathogen:** microorganisms typically belonging to the skin microbiota (Coagulase-Negative Staphylococci, (CoNS), *Micrococcus* spp., *Bacillus* spp., *Corynebacterium* spp., and *Propionibacterium* spp.) were considered to be probable contaminants and excluded [21] An exception to this was CoNS isolated from blood cultures taken from at least two different puncture sites on the same occasion.

**Repeat isolate:** culture of same species with identical susceptibility pattern isolated during the same admission episode (from admission until hospital discharge). Repeat isolates were excluded.

**Episodes of BSI:** an episode fulfilling the criterion “positive blood culture with a significant pathogen”. If a patient had more than one BSI-episode per admission, only the first BSI-episode was included in the analysis involving comorbidity and mortality.

**Hospital-acquired bloodstream infection (HA-BSI):** BSI occurring ≥ two calendar days after hospital admission.

**Community-onset bloodstream infection (CO-BSI):** BSI occurring < two calendar days from admission.

**New episode of BSI:** infection caused by a different bacterial or fungal pathogen >3 calendar days after the previous admission or by the same bacterial or fungal pathogen >30 calendar days after the previous admission.

**Comorbidity:** We selected comorbidities based on the EPIC-II protocol [22] and adapted these to the ICD code system. We made a few modifications/clarifications to the EPIC II protocol for certain comorbidities (see below). Steroid treatment was excluded because data for this could not be retrieved from the register. Obesity was not included in the EPIC II protocol and was thus not considered in our study. Data on diagnoses with one of the following ICD-10 codes documented in the patient´s journal up to 24 months prior to date of admission were included (EPIC II): chronic obstructive pulmonary disease (COPD) (ICD:J44) (EPIC II COPD); chronic renal failure (ICD:N18) (EPIC II chronic renal failure); diabetes mellitus (ICD:E10-E14) (EPIC II insulin dependent diabetes mellitus); haematologic malignancy (ICD:C81-C96) (EPIC II hematologic cancer); diseases of the blood and blood-forming organs, and certain disorders involving the immune mechanism (ICD:D50-D89); and immunosuppression (ICD:D80-D89) and transplant patient (ICD:Z94.0-Z94.6, Z94.8-Z94.9) (EPIC II immunosuppression); tumour (ICD:C00-D48) (EPIC II cancer, metastatic cancer); chronic liver disease + cirrhosis (ICD:K74, B18, K70.3, K71.7); HIV (ICD:B20-B24) (EPIC II cirrhosis); HIV (ICD:B20-B24) (EPIC II HIV) heart failure (ICD:I20-I25) (EPIC II heart failure, NYHA III-IV).

### Antibiotic Consumption

All drug statistics were collected from national drug consumption statistics published by the Swedish e-Health Agency and accessed through a dedicated secure web-site [23]. Consumption of systematic antibiotics (defined either as J01 in the ATC-code for international comparison, or J01, excluding J01XX05 methenamine as the national Swedish quality indicator) was measured as defined daily doses (DDD) per 1,000 inhabitants and day. Dispensed prescriptions were measured per 1,000 inhabitants and year. Use of systemic antibiotic consumption in hospital was measured as DDD using episode of care or days of care as denominators. [23, 24]

### Ethics approval

The study was approved by the Linköping Regional Ethics Committee (2010/160-31).

### Statistical Analyses

We assessed changes in annual incidence of BSI (HA and CO) using Poisson regression, presenting the change in incidence as a ratio (IRR) with a 95% confidence interval (CI). To determine whether differences in clinical characteristics between HA and CO were statistically significant, we used the Wilcoxon rank-sum test and chi-square test. The predominant pathogens were defined at genus or species level, and annual trends were tested using linear regression. Chi-square and t-tests were used for univariate analyses to determine risk factors for BSI and 30-day mortality. Multivariable binomial regression analysis adjusted for confounding was used to determine risk factors. The following variables were used in the regression model and investigated as possible independent risk factors for BSI: gender; age; comorbidity; and year of diagnosis. A patient without infection in this context was a patient with a negative blood cultures. The same variables plus BSI (patients with HA-BSI or CO-BSI compared to those without infection) were investigated as risk factors for 30-day mortality. A p-value lower than 0.05 was considered significant. All statistical analyses were performed with Stata version 13.1 (StataCorp LP, College Station, TX, USA in Linköping, Sweden).

## Results

### Clinical Characteristics of the Study Population

Over the 14-year period, the total number of admissions increased from 73,410 to 75,214 and the population from 411,345 to 437,848, but the number of admissions per 1,000 inhabitants decreased from 178 to 172 (p = 0.25). The average hospital stay decreased from 5.7 to 4.4 days of care (p<0.01), and the average median age decreased from 59 to 58 years (p = 0.34) in all hospitalised patient (Data A in S1 File). Over the years 2000–2013, the median age of all patients with a positive blood culture, increased from 67 to 69 years for HA-BSI (p = 0.34) and from 67 to 68 for CO-BSI (p = 0.14), (Data A in S1 File). During the study period, 11,480 episodes of BSI were detected, of which 3,788 were HA-BSI (33%) and 7,692 (67%) were CO-BSI. Characteristics of patients with CO-BSI and HA-BSI are shown in Table 1.

**Table 1:** Characteristics of patients with CO-BSI and HA-BSI.

		BSI (n = 11,480)***	CO-BSI (n = 7,692)	HA-BSI (n = 3,788)	P-value CO vs HA	
**Demographics**						
Age, median (25–75 percentile)		71 (58–81)	73 (60–82)	68 (55–78)	<0.001 *	
<25		544 (5%)	321 (4%)	223 (6%)		
25–65		3791 (33%)	2317 (30%)	1474 (39%)		
66–80		4087 (36%)	2718 (35%)	1369 (36%)		
>80		3058 (27%)	2336 (30%)	722 (19%)		
Female		5042 (44%)	3482 (45%)	1560 (41%)		
Men		6438 (56%)	4210 (55%)	2228 (59%)	<0.001 **	
**Type of admission**						
Medical		**8,301 (72%)**	**6,077 (79%)**	**2,224 (59%)**		
	Paediatrics	271 (2.4%)	162 (2.1%)	109 (2.9%)		
	Oncology	152 (1.3%)	57 (0.7%)	95 (2.5%)		
	Haematology	720 (6.3%)	90 (1.2%)	630 (16.6%)		
	Infection	831 (7.2%)	708 (9.2%)	123 (3.2%)		
	General medicine	2,948 (25.7%)	2,418 (31.4%)	530 (14%)		
	Geriatric	146 (1.3%)	53 (0.7%)	93 (2.5%)		
	Emergency department	2,083 (18.1%)	1,884 (24.5%)	199 (5.3%)		
	Other	1,150 (10%)	705 (9.2%)	445 (11.7%)		
Surgical		**2,215 (19%)**	**1,018 (13%)**	**1,197 (32%)**		
	General surgery	1332 (11.6%)	704 (9.2%)	628 (16.6%)		
	Thoracic surgery	150 (1.3%)	26 (0.3%)	124 (3.3%)		
	Plastic and hand surgery	125 (1.1%)	8 (0.3%)	117 (3.1%)		
**Neurosurgery**		102 (0.9%)	12 (0.2%)	90 (2.4%)		
	Orthopaedics and back surgery	143 (1.2%)	52 (0.7%)	91 (2.4%)		
	Urology	323 (2.8%)	188 (2.4%)	135 (3.6%)		
	Other	40 (0.3%)	28 (0.4%)	12 (0.3%)		
ICU		**400 (3.5%)**	**160 (2%)**	**240 (6.3%)**		
Other		**549 (4.8%)**	**425 (5.5%)**	**124 (3.3%)**		
**Hospital**						
Linköping (Tertiary care)		5,820 (50.7%)	3,415 (44.4%)	2,405 (63.5%)	<0.001 **	
Norrköping (Secondary care)		3,814 (33.2%)	2,799 (36.4%)	1,015 (26.8%)		
Motala (Secondary care)		1,400 (12.2%)	1,111 (14.4%)	289 (7.6%)		
Finspång (Primary care)		91 (0.8%)	69 (0.9%)	22 (0.6%)		
Not specified		355 (3.1%)	298 (3.9%)	57 (1.5%)		
**Comorbidity**	**ICD**		**CO-BSI (n = 7,692)**	**HA-BSI (n = 3,788)**		
COPD	J44		401 (5.2%)	173 (4.6%)		0.135 **
Chronic renal failure	N18		363 (4.7%)	203 (5.4%)		0.137 **
Diabetes mellitus	E10-E14		1,457 (18.9%)	605 (16.0%)		<0.001 **
Diabetes mellitus	E10		259 (3.4%)	132 (3.5%)		0.744 **
Haematologic malignancy	C81-C96		258 (3.4%)	407 (10.7%)		<0.001 **
Transplant patient	Z94 (Z94.0 –Z94.9) Excluding Z94.7		106 (1.4%)	75 (2.0%)		0.015 **
Tumour	C00-D48		1,167 (15.2%)	1,097 (29.0%)		<0.001 **
Chronic liver disease + Cirrhosis	K74, B18, K70.3, K71.7		215 (2.8%)	87 (2.3%)		0.117 **
HIV	B20-B24		6 (0.1%)	2 (0.1%)		0.630 **
Heart failure	I20-I25		908 (11.8%)	470 (12.4%)		0.350 **
Diseases of the blood and blood-forming organs and certain disorders involving the immune mechanism	D50-D89		591 (7.7%)	430 (11.4%)		<0.001 **
Immunosuppression	D80-D89		20 (0.3%)	16 (0.4%)		0.143 **
**Number of comorbidities**						
0			3,978 (51.7%)	1,633 (43.1%)		<0.001 **
1			2,247 (29.2%)	1,048 (27.7%)		
2			1,012 (13.2%)	770 (20.3%)		
>2			455 (5.9%)	337 (8.9%)		
**Outcome**						
30-day mortality		1464 (12.8%)	814 (10.6%)	650 (17.2%)		<0.001 **
Hospital length of stay (median days (25–75 percentile)			5 (3–10)	10 (5–20)		<0.001 *

* Wilcoxon rank-sum test

** Chi square test

*** Unique episode of BSI

### Microbiology

Over the study period, 14,010 microorganisms were obtained from blood cultures, yielding 11,480 BSI episodes (Fig 1). The proportion of hospital admissions where a blood culture was taken increased from 8.2 to 15.4% (6,064 to 11,632) (p<0.01). The proportion of hospital admissions with a positive blood culture increased from 1.1 to 1.7% (p<0.01), though there was a minor decrease in the proportion of blood cultures that were positive (from 12.9 to 11.3%) (p = 0.02) (Data A and B in S1 File). Yearly distributions of species are shown in Table 2 and Data C in S1 File.

**Table 2:** Distribution of most common blood isolates.

																	Linear regression		
	2000***	01	02	03	04	05	06	07	08	09	10	11	12	2013	Total	Change %*	Average yearly increase**	95% CI	P-value
**Gram negative**	**339**	**342**	**333**	**350**	**356**	**351**	**385**	**473**	**405**	**493**	**620**	**593**	**693**	**710**	**6,443**	**104%**	**39.6**	**30.52–48.67**	**<0.01**
** Other than Enterobacteriacae**	**40**	**28**	**30**	**38**	**36**	**37**	**47**	**48**	**43**	**65**	**52**	**54**	**67**	**76**	**661**	**85%**	**3.9**	**2.68–5.18**	**<0.01**
** ***Acinetobacter baumannii*	1	0	1	1	0	0	0	1	0	1	4	2	1	5	17	371%	0.3		
** ***Pseudomonas aeruginosa*	18	12	9	14	14	15	24	15	20	27	20	22	28	25	263	36%	1.4		
** ***Haemophilus influenzae*	5	5	12	7	5	4	2	6	6	6	3	8	14	7	90	37%	0.2		
** Enterobacteriaceae**	**269**	**290**	**283**	**295**	**296**	**293**	**311**	**397**	**338**	**388**	**535**	**505**	**579**	**604**	**5,383**	**119%**	**34**	**25.45–42.62**	**<0.01**
** ***Escherichia coli*	171	180	183	172	173	167	174	237	192	255	322	301	355	395	3277	126%	21.5		
** ***Klebsiella pneumoniae*	31	37	43	41	42	40	41	61	48	38	85	74	92	69	742	117%	4.9		
** ***Klebsiella oxytoca*	15	17	16	16	20	16	16	25	23	17	27	33	33	33	307	115%	1.9		
** ***Enterobacter cloacae*	12	18	17	24	26	23	17	12	29	23	28	25	38	22	314	79%	1.3		
**Gram positive**	**472**	**463**	**426**	**450**	**494**	**466**	**476**	**511**	**451**	**579**	**537**	**532**	**680**	**696**	**7,233**	**44%**	**19.7**	**11.25–28.08**	**<0.01**
** Enterococci**	**49**	**70**	**64**	**74**	**63**	**47**	**60**	**77**	**53**	**94**	**73**	**69**	**96**	**104**	**993**	**107%**	**3.4**	**0.86–5.91**	**0.01**
** ***Enterococcus faecalis*	32	43	47	44	39	31	37	47	35	57	50	40	58	62	622	89%	1.8		
** ***Enterococcus faecium*	15	18	10	24	17	9	21	28	16	33	14	20	26	33	284	115%	1.3		
** Staphylococci**	**199**	**199**	**176**	**161**	**214**	**195**	**198**	**223**	**188**	**216**	**235**	**172**	**269**	**270**	**2,915**	**32%**	**5.7**	**0.62–10.81**	**0.03**
** ***Staphylococcus aureus*	142	130	115	128	139	140	144	166	145	167	211	157	256	258	2298	77%	11.4		
** ***Coagulase-Negative Staphylococci*	57	69	61	33	72	48	50	49	33	40	13	8	8	10	551	-83%	-6.4		
** Streptococci**	**152**	**138**	**144**	**154**	**168**	**157**	**162**	**148**	**162**	**197**	**177**	**207**	**213**	**223**	**2,402**	**43%**	**7.2**	**4.86–9.45**	**<0.01**
** ***Streptococcus pneumoniae*	52	53	53	63	60	65	65	46	59	72	53	65	52	50	808	-6%	-0.1		
** ***Streptococcus pyogenes (A)*	23	13	7	14	18	11	13	13	10	20	10	20	23	23	218	-2%	0.6		
** ***Streptococcus agalactiae (B)*	12	12	11	13	4	10	13	9	21	12	18	18	19	22	194	79%	1.1		
** ***Streptococcus spp*. *(C*, *G)*	7	11	9	10	14	19	15	24	23	25	30	24	38	34	283	376%	2.9		
** Yeast**	**17**	**20**	**14**	**11**	**15**	**27**	**12**	**32**	**16**	**20**	**33**	**44**	**39**	**28**	**328**	**60%**	**2.3**	**0.71–3.82**	**0.01**
** ***Candida albicans*	10	14	7	8	9	15	5	20	11	11	22	27	17	17	193	66%	1.2		
** ***Candida spp (non albicans)*	7	6	7	3	6	12	7	12	5	9	11	17	22	11	135	57%	1.1		

* Change in rate from 2000–2013 per 100,000 hospital admissions

** Increased cases per year per 100,000 hospital admissions, (average annual increase %)

*** Hospital admissions, 2000 (73,410), 2001 (73,395), 2002 (71,791), 2003 (71,945), 2004 (68,954), 2005 (68,454), 2006 (70,586), 2007 (69,982), 2008 (69,363), 2009 (72,383), 2010 (72,766), 2011 (73,849), 2012 (75,516), 2013 (75,214)

### Trends in episodes of BSI

The incidence of BSI, CO-BSI and HA-BSI increased by 64%, 83% and 32% respectively with hospital admissions as denominator (Table 3). Corresponding figures with hospital days as the denominator were; 111%, 129% and 70% respectively (Data D in S1 File).

**Table 3:** Incidence rate of BSIs (HA and CO) 2000–2013 per 100,000 hospital admissions.

																Linear regression		
	2000	2001	2002	2003	2004	2005	2006	2007	2008	2009	2010	2011	2012	2013	Change %*	Average yearly increase**	*95%* CI	P-value
**BSI**	945	928	911	912	1043	1030	1024	1167	1018	1205	1351	1323	1496	1546	64%	48 (3.9%)	35.22–60.45	<0,01
**HA**	357	372	313	343	332	329	315	400	336	391	427	418	444	471	32%	9	4.25–14.22	<0,01
**CO**	589	556	598	569	711	701	710	767	682	814	924	905	1053	1076	83%	39	29.69–47.50	<0,01

* Change in rate from 2000–2013 per 100.000 hospital admissions

** Increased cases per year per 100,000 hospital admission, (average annual increase, was calculated, ex BSI, 1.64 √13)

### Antibiotic Consumption

During the period studied the amount of systemic antibiotics agents consumed in outpatient care remained largely unchanged at an average of 12.9 defined daily doses (DDD) per 1,000 inhabitants and day (range 11.7–13.5). However, the number of dispensed prescriptions decreased 24% from 422 to 322 per 1,000 inhabitants and year (Data E in S1 File). The total amount of systemic antibiotics (J01) consumed in hospital increased by 32.8% from 177,704 DDD to 235,985 DDD. This corresponds to an increase of 29.8% in DDD per episode of care (2.42 to 3.14 DDD per episode of care), or 67.2% of DDD per day of care (424 to 709 DDD per 1,000 days of care) (Data F and G in S1 File). [23]

### Comorbidity—Univariate Analysis

**Fig 2:**
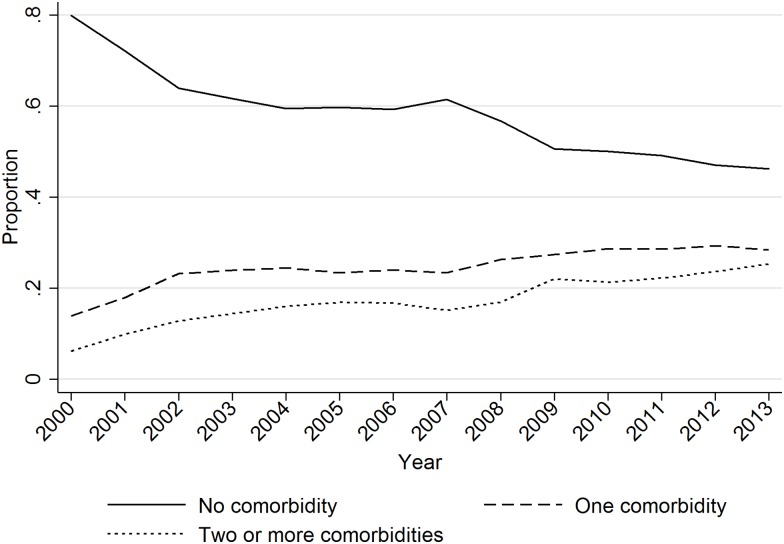
Comorbidities among blood cultured patients.

HA-BSI was significantly more common in patients who also had haematologic malignancy (HA 10.7% vs CO 3.4%), tumour (HA 29.0% vs CO 15.2%); diseases of the blood and blood-forming organs; e.g. anaemias (HA 11.4% vs CO 7.7%). Diabetes mellitus was significantly more common in patients with CO-BSI (HA 16.0% vs CO 18.9%). The proportions of co-morbidity among patients with BSI are shown in Table 1 and Fig 2.

### Mortality—Univariate Analysis

**Fig 3:**
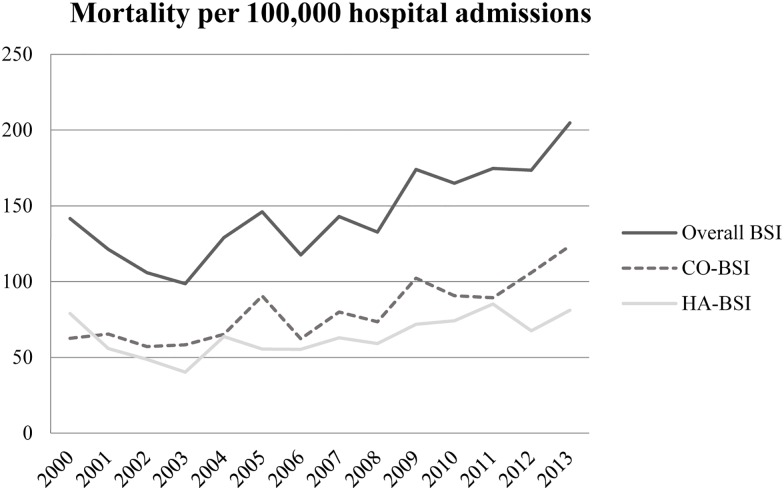
Mortality per 100,000 hospital admissions.

The 30-day mortality rate amongst patients with a positive blood culture increased from 142 per 100,000 hospital admissions per year in 2000 to 205 in 2013, an increase of 44.5% (average annual increase 2.9%) (p<0.01). Death within thirty days among patients with HA-BSI increased from 79 to 81 (2.7% increase) (p = 0.05) and among those with CO-BSI from 63 to 124 per 100,000 admissions per year (97.1% increase) (p = <0.01) (Fig 3 and Data H).

Over the study period a total of 1,464 patients died within 30 days of the date when the blood culture was taken (650 with HA-BSI and 814 with CO-BSI), giving an overall 30-day mortality of 17.2% for HA-BSI and 10.6% for CO-BSI (p<0.01) (Table 1).

### Multivariable risk factor analyses

In multivariable analyses, we found the following risk factors for 30-day mortality: HA-BSI, with patients with negative blood culture as reference (RR = 2.22, 95% confidence interval: 2.02–2.33); two or more comorbidities (RR = 1.89, 95% CI: 1.79–2.00); one comorbidity (RR = 1.56, 95% CI: 1.48–1.65); CO-BSI, with patients with negative blood culture as reference (RR = 1.21, 95% CI: 1.13–1.30); male gender (RR = 1.05, 95% CI: 1.01–1.11); and age (RR = 1.04, 95% CI: 1.04–1.05) (Table 4).

**Table 4:** Risk factors for 30-day mortality.

30-day mortality			
	Risk Ratio*	95% -Conf	P-value
**CO-BSI**	1.21	1.13–1.3	<0.01
**HA-BSI**	2.22	2.02–2.33	<0.01
**Age**	1.04	1.04–1.05	<0.01
**Female**	0.95	0.91–0.99	0.02
**Year**	0.98	0.98–0.99	<0.01
**Comorbidity**			
**1**	1.56	1.48–1.65	<0.01
**≥2**	1.89	1.79–2	<0.01

* Multivariate binomial regression analysis

## Discussion

This survey revealed a surprisingly large overall increase in incidence of BSI, as well as increased crude mortality and comorbidity among patients with BSI. Comorbidity was an independent risk factor for both BSI and mortality. The incidence of CO-BSI increased by 83% from 2000 to 2013. There are several possible explanations: more vulnerable patients which are supported by the increased incidence of co-morbidity, a higher awareness of sepsis, supported by the increased number of blood cultures, different patient groups or factors outside the hospital such as delay to appropriate treatment. The results of this study have initiated a “sepsis alarm” programme in prehospital care, emergency units and the four hospitals in the county.

The most prominent increase in BSI was due to *E*. *coli*. A similar trend, with an increase in *E*. *coli* bacteraemia, has been reported from the UK where in 2008, 23% of all cases of bacteraemia were caused by this species [25].

We excluded episodes of BSI with probable skin contaminants (CoNS, *Micrococcus* spp., *Bacillus* spp., *Corynebacterium* spp., *Propionibacterium* spp.), as in the study by Ammerlaan et al [21]. However, if *CoNS* was isolated from blood culture samples from at least two different puncture sites taken on the same occasion, then this was registered as a BSI since these may have been due to central line-associated BSI (CLABSI). In our study, 3.9% of all BSIs were caused by *CoNS* compared to 10% in the study by Skogberg et al that did not exclude skin contaminants, and 10% in the study by Nielsen et al including skin contaminants if detected in ≥ two blood culture sets within 5 days [26, 27]. Comparisons with BSI studies not excluding skin contaminants should be made with caution, since this may have an impact on most data including incidence, infectious agent and mortality.

We did not assess the number of healthcare-associated BSIs (HCA-BSI) acquired in long-term care facilities—since it was not possible to separate these patients from patients with true community-onset BSI using data from the patient-administration system. This limitation was also seen in the Danish BSI study by Nielsen et al, with 46% community-acquired, 31% nosocomial and 23% healthcare-associated BSIs [26]. The latter group included only patients discharged from an outpatient clinic in haematology, nephrology, or oncology within 30 days prior to the admission. The incidence of 31% nosocomial BSIs reported in the Danish study is similar to the 33% hospital-acquired (HA)-BSI in our study. Only 2% of patients registered as CO-BSI were admitted to a haematology or oncology department, but among the patients with HA-BSI 19% were treated on these wards indicating that amongst patients with cancer, most BSIs were registered as HA-BSI. However, the exact proportion of healthcare-associated but not hospital-acquired BSIs among those with community-onset BSI (CO-BSI) cannot be ascertained from our data. Patients with HA-BSI had a significantly longer hospital stay compared to those with CO-BSI, which is partially a consequence of the definition used and is in agreement with other studies [28, 17, 29, 30].

Another potential limitation of our study is that some postoperative BSIs may have been misclassified as CO-BSI but were, in fact, HA-BSI, although blood cultures were taken <2 days after admission. However, HA-BSI was slightly more common than CO-BSI on surgical wards, which would suggest that misclassification might not have influenced the results to any great extent.

There are several factors that may explain the increase in BSI. One contributing factor might have been the increased numbers of blood cultures taken per patient admitted (from 8.2% to 15.4% per hospital admission), as a result of the surviving sepsis campaign. The fact that the fraction of blood cultures that were positive only decreased slightly (from 12.9 to 11.3%) indicates that no major change in strategy for ordering blood cultures took place during the study period.

The increase in blood cultures taken per patient was also observed in a study by Skogberg et al [12]. Our findings showing increased incidence of BSI and increased crude mortality are in agreement with those of a recent Finnish study by Skogberg et al 2012, which showed annual increases in BSI and mortality of 4.4%, and 4.0% respectively [10]. In contrast, a Danish study by Nielsen et al reported an annual decrease in BSI of 3.3% between 2000 and 2008. Despite this decrease in BSI there was still a high overall incidence rate of 215.7 BSI per 100,000 persons per year in 2008.

The incidence of BSI has increased in most other population-based studies [11, 8, 27, 9], but the range is wide in published studies (76 to 215 BSI per 100,000 persons per year) [17, 8, 31, 32, 27, 33]. Since the incidence in this study increased from 169 BSI/100,000 persons/year in 2000 to 265 BSI/100,000 persons/year in 2013 we are above the upper range compared to these published studies.

Since this is a retrospective study based on database information, we cannot rule out the possibility that changes in classification and reporting of diagnoses took place during the study period that could partly explain the increased comorbidity. A limitation is the absence of exact time for admission and BSI samplings, plus the fact that the minimum actual hours a patient could be hospitalised before a BSI was defined as an HA-BSI according to our study (> two calendar days, see method) could have been 25 hours. However, the mean time patients with HA-BSI had their blood culture taken was 6.3 days after admission, which would suggest that this might not have influenced the classification of HA-BSI to any great extent. This is also supported by the corresponding mean time for CO-BSI, which was 0.12 days from admission.

According to a recent study, public awareness of sepsis is low in Sweden [34] which may contribute to patient delay in contacting the healthcare services. The higher incidence of BSI seen in men and individuals with comorbidity, seen in this survey, is in agreement with previous studies [35, 36, 26]

Several educational measures to reduce antibiotic use in the community have been implemented over the last 20 years in Östergötland county as a part of the Swedish Strama antibiotic stewardship programme [37]. A key component of this programme is to reduce unnecessary outpatient use of antibiotics. A national target of 250 dispensed prescriptions of antibiotics per 1,000 inhabitants per year was set in 2010 based on an estimation of possible overuse in another county [38–40]. During 2011–2014 the government introduced financial incentives for counties that reduced their use of antibiotics. During the study period the use of antibiotics on prescription decreased by 24% to 322 dispensed prescriptions per 1,000 inhabitants per year, whilst the amount measured in defined daily doses per 1,000 inhabitants per day remained relatively unchanged.

Since, the causal relationship between comorbidity and increased incidence of BSI is not clear, we suggest further studies to investigate this and other possible causes of increased BSI and BSI related mortality, such as low public awareness of sepsis, late detection and treatment of sepsis in pre-hospital healthcare, restrictive antibiotic prescription, and increased frequency of infections caused by antibiotic-resistant bacteria. Sweden is a country with low overall antibiotic resistance [41, 42]. Thus, treatment failure due to infection with antibiotic-resistant bacteria was probably not common since the resistance rate for major pathogens was low. Hence, antibiotic resistance probably did not have any major effect on mortality in this study. In contrast, the study by Ammerlaan et al, mainly conducted in European hospitals and using basically the same BSI definitions as in our study, showed that nosocomial BSIs due to antibiotic-resistant bacteria increased [21]. Similar findings have been reported from a large study in the US [5, 4]. Sweden has one of Europe’s lowest outpatient antibiotic consumption [43]. It is not known how this influences the incidence of BSI since there is no national BSI register. Antibiotic use in the outpatient setting decreased from 2010, despite the increased burden of community onset BSI but this study was not designed to investigate any possible connection. We do not know the number of patients with CO-BSI seeking medical attention at the hospital emergency department after first attending a primary care unit or hospital outpatient clinic or after receiving advice over the phone from a qualified healthcare advisor at the Swedish telemedicine-support service. Thus, we cannot determine the number of BSI cases with non-appropriate pre-hospital care, but this will be analysed before and after multiple interventions for improved care of the sepsis-patient which will start at the end of 2016. Furthermore, since this is a population-based study without data from each patient´s medical records, we could not determine if there actually was under-treatment with antibiotics in out-patient care which resulted in the 69% increase in hospital antibiotic use expressed as DDD per day of care. Interestingly the most common species *E*. *coli* and *S*. *aureus* increased by 126% and 77% respectively, whereas *S*. *pneumoniae* and Streptococcus pyogenes decreased 6 and 2% respectively. The reasons for the increase in incidence of both *E*.*coli* and *S*.*aureus* bacteraemia will be explored in a prospective study recently started by our group.

Interventions to decrease BSI is crucial in our healthcare setting and multi-target interventions in a “sepsis alarm” programme, covering prehospital care, emergency units and hospital care, will be introduced at the end of 2016. All patients will be triaged in the ambulance and at the emergency units for sepsis using broad sepsis criteria including, but not limited to, the new qSOFA score [44], and a focused treatment and follow-up programme using among other algorithms the surviving sepsis protocol [2]. The purpose is also to implement a treatment and follow-up protocol enabling early intensive treatment for those with confirmed sepsis, and early de-escalation of treatment for those who are subsequently diagnosed with a non-sepsis ailment. In addition our BSI register will be a part of the “sepsis alarm” programme. The BSI register will be used for “crash investigation” of all fatal cases of BSI with the aim to find preventable risk factors for mortality associated with BSI, and to improve our antibiotic stewardship programme.

In conclusion, this study, performed in a Swedish setting, showed increases in the incidence of BSI and BSI-related crude mortality over a 14-year period (2000–2013). An increase in comorbidity in BSI patients was also observed, and was an independent risk factor for both BSI and mortality. It is essential to detect BSI among patients with underlying disease at an early stage and to improve antibiotic therapy. Interventions to decrease BSI in general should be considered together with robust antibiotic stewardship programmes to avoid both over- and underuse of antibiotics.

## Supporting Information

S1 File Blood culture characteristics (hospital admission, length of stay, age, blood cultures, and microorganism) (Data A), Blood culture characteristics (blood culture per hospital admission and days, hospital admission per 1,000 inhabitants, positive blood culture per total number of blood cultures) (Data B), Distribution of common microorganisms causing BSI, in south-east Sweden, 2000–2013 (Data C), Incidence rate of BSIs (HA and CO) 2000–2013 per 100,000 hospital days (DataD), Antibacterial for systemic use excluding metenamine (J01-J01XX05) dispensed to outpatients 2000–2015 measured as defined daily doses per 1,000 inhabitants and day, or dispensed prescriptions per 1,000 inhabitants and year (Data E) Amount of systemic antibiotics (J01) used on hospital wards and outpatient clinics measured in defined daily doses (DDD) per 1,000 hospital day (Data F), Amount of systemic antibiotics (J01) used on hospital wards and outpatient clinics measured in defined daily doses (DDD) per episode of care. (Data G), Mortality per 100,000 hospital admission (BSI, HA, CO) (Data H), Bloodstream infection—incidence per hospital admission (adjusted for, sex, age and comorbidities Poisson regression). (Data I), Mortality—Incidence per hospital admission (adjusted for, sex, age and comorbidities Poisson regression). (Data J), Comorbidities among blood cultured patients (n = 109,983) (Data K), Quality indicators of systemic antibiotics (J01) for outpatient use as defined by ESAC (European Surveillance of Antimicrobial Consumption) REF2007. Measured in defined daily doses per 1,000 inhabitants and day; or percentage of total amount of J01 measured in DDD, or as specified fraction measured in DDD. (Data L).
(DOCX)
