# Supplementary material for: 14-Year Survey in a Swedish County Reveals a Pronounced Increase in Bloodstream Infections (BSI). Comorbidity - An Independent Risk Factor for Both BSI and Mortality
Source: PLoS One. 2016 Nov 11;11(11):e0166527. doi: 10.1371/journal.pone.0166527 (PMC5106013; doi:10.1371/journal.pone.0166527)
Supplement: S1 File — Blood culture characteristics (hospital admission, length of stay, age, blood cultures, and microorganism) (Data A), Blood culture characteristics (blood culture per hospital admission and days, hospital admission per 1,000 inhabitants, positive blood culture per total number of blood cultures) (Data B), Distribution of common microorganisms causing BSI, in south-east Sweden, 2000–2013 (Data C), Incidence rate of BSIs (HA and CO) 2000–2013 per 100,000 hospital days (DataD), Antibacterial for systemic use excluding metenamine (J01-J01XX05) dispensed to outpatients 2000–2015 measured as defined daily doses per 1,000 inhabitants and day, or dispensed prescriptions per 1,000 inhabitants and year (Data E) Amount of systemic antibiotics (J01) used on hospital wards and outpatient clinics measured in defined daily doses (DDD) per 1,000 hospital day (Data F), Amount of systemic antibiotics (J01) used on hospital wards and outpatient clinics measured in defined daily doses (DDD) per episode of care. (Data G), Mortality per 100,000 hospital admission (BSI, HA, CO) (Data H), Bloodstream infection—incidence per hospital admission (adjusted for, sex, age and comorbidities Poisson regression). (Data I), Mortality—Incidence per hospital admission (adjusted for, sex, age and comorbidities Poisson regression). (Data J), Comorbidities among blood cultured patients (n = 109,983) (Data K), Quality indicators of systemic antibiotics (J01) for outpatient use as defined by ESAC (European Surveillance of Antimicrobial Consumption) REF2007. Measured in defined daily doses per 1,000 inhabitants and day; or percentage of total amount of J01 measured in DDD, or as specified fraction measured in DDD. (Data L). (DOCX) [file pone.0166527.s001.docx]

**Supplementary data (S1 File)**

**Data A.** Blood culture characteristics (hospital admission, length of stay, age, blood cultures, and microorganism)

|  |  |  |  |  |  |  |  |  |  |  |  | | |
| --- | --- | --- | --- | --- | --- | --- | --- | --- | --- | --- | --- | --- | --- |
| **Year** | **Hospital admission** | **Hospital days** | **Average length of stay (days median)*** | **Age, median of all hospitalised patient**** | **Age, median of all patient with blood culture obtained*****  **HA CO** | | **Numbers of all blood cultures** | **Positive blood cultures** | **Micro-organisms ****** | **Population in Östergötland** | **BSI- Total** | **HA-BSI** | **CO-BSI** |
| **2000** | 73 410 | 418 758 | 6 | 59 | 67 | 67 | 6064 | 782 | 828 | 411 345 | 694 | 262 | 432 |
| **2001** | 73 395 | 400 938 | 5 | 59 | 69 | 65 | 6066 | 755 | 825 | 412 363 | 681 | 273 | 408 |
| **2002** | 71 791 | 399 940 | 6 | 59 | 67 | 67 | 6132 | 729 | 775 | 413 438 | 654 | 225 | 429 |
| **2003** | 71 945 | 368 272 | 5 | 58 | 67 | 67 | 6185 | 755 | 811 | 414 897 | 656 | 247 | 409 |
| **2004** | 68 954 | 354 861 | 5 | 58 | 68 | 67 | 6055 | 808 | 865 | 415 990 | 719 | 229 | 490 |
| **2005** | 68 454 | 340 337 | 5 | 59 | 68 | 68 | 6463 | 788 | 845 | 416 303 | 705 | 225 | 480 |
| **2006** | 70 586 | 331 436 | 5 | 59 | 68 | 66 | 6601 | 814 | 873 | 417 966 | 723 | 222 | 501 |
| **2007** | 69 982 | 341 580 | 5 | 59 | 68 | 66 | 7548 | 923 | 1016 | 420 809 | 817 | 280 | 537 |
| **2008** | 69 363 | 378 999 | 5 | 58 | 67 | 67 | 7860 | 793 | 873 | 423 169 | 706 | 233 | 473 |
| **2009** | 72 383 | 324 711 | 4 | 58 | 68 | 67 | 8710 | 1022 | 1093 | 427 106 | 872 | 283 | 589 |
| **2010** | 72 766 | 337 520 | 5 | 58 | 67 | 67 | 9211 | 1100 | 1190 | 429 642 | 983 | 311 | 672 |
| **2011** | 73 849 | 330 589 | 4 | 59 | 68 | 66 | 10062 | 1102 | 1170 | 431 075 | 977 | 309 | 668 |
| **2012** | 75 516 | 332 719 | 4 | 59 | 68 | 69 | 11394 | 1264 | 1412 | 433 784 | 1130 | 335 | 795 |
| **2013** | 75 214 | 332 745 | 4 | 58 | 69 | 68 | 11632 | 1314 | 1434 | 437 848 | 1163 | 354 | 809 |
| **Total/ p-value** |  |  | <0.01 | =0.34 | =0.34 | =0.14 | 109983 | 12949 | 14010 |  | 11480 | 3788 | 7692 |

* Average length of stay (median days) for all patient hospitalised in Ostergotland 2000-2013.

** Median age (25-75 percentiles) all hospitalised patients even patients without bloodstream infection 2000-2013.

*** Median age (25-75 percentiles) all patients from whom the blood culture was obtained 2000-20013

****Microorganism (bacterial and yeast isolates) typically belonging to the skin microbiota (coagulase-negative-Staphylococci (CoNS), *Micrococcus* spp, *Bacillus spp*, *Corynebacterium* spp, *Propionibacterium spp*.) were considered to be probable contaminants and were excluded , with one exception; *CoNS* isolated in at least two different puncture sites of blood cultures taken on the same day.

**Data B.** Blood culture characteristics (blood culture per hospital admission and days, hospital admission per 1,000 inhabitants, positive blood culture per total number of blood culture)

|  | |  |  |  |  |  |  |  |
| --- | --- | --- | --- | --- | --- | --- | --- | --- |
| Year | **Blood cultures per hospital admission*** | **Blood cultures per hospital days**** | **Hospital admission per 1,000 inhabitants** | **Positive blood cultures per total numbers of blood cultures** | **Positive blood cultures per hospital admission** | **Positive blood cultures per hospital days** | **BSI per 100,000 hospital admissions** | **BSI per 100,000 hospital days** |
| 2000 | 8.2% | 1.4% | 178 | 12.9% | 1.1% | 0.2% | 945 | 166 |
| 2001 | 8.3% | 1.5% | 178 | 12.4% | 1.0% | 0.2% | 928 | 170 |
| 2002 | 8.5% | 1.5% | 174 | 11.9% | 1.0% | 0.2% | 911 | 164 |
| 2003 | 8.6% | 1.7% | 173 | 12.2% | 1.0% | 0.2% | 912 | 178 |
| 2004 | 8.8% | 1.7% | 166 | 13.3% | 1.2% | 0.2% | 1043 | 203 |
| 2005 | 9.4% | 1.9% | 164 | 12.2% | 1.2% | 0.2% | 1030 | 207 |
| 2006 | 9.3% | 2.0% | 169 | 12.3% | 1.2% | 0.2% | 1024 | 218 |
| 2007 | 10.8% | 2.2% | 166 | 12.2% | 1.3% | 0.3% | 1167 | 239 |
| 2008 | 11.4% | 2.1% | 164 | 10.1% | 1.1% | 0.2% | 1018 | 186 |
| 2009 | 12.2% | 2.7% | 169 | 11.7% | 1.4% | 0.3% | 1205 | 269 |
| 2010 | 12.8% | 2.8% | 169 | 11.9% | 1.5% | 0.3% | 1351 | 291 |
| 2011 | 13.6% | 3.0% | 171 | 11.0% | 1.5% | 0.3% | 1323 | 296 |
| 2012 | 15.1% | 3.4% | 174 | 11.1% | 1.7% | 0.4% | 1496 | 340 |
| 2013 | 15.4% | 3.5% | 172 | 11.3% | 1.7% | 0.4% | 1546 | 350 |
| P-value*** | <0,01 | <0.01 | 0.25 | 0.02 | <0.01 | <0.01 | <0.01 | <0.01 |
| Change (%)**** | 87% | 150% | -3.4% | -12% | 55% | 100% | 64% | 111% |

*All blood cultures per hospital admissions

** All blood cultures per hospital days

***P-value for trend (2000-2013)

****Change (%) 2000vs2013

**Data C.** Distribution of most commonly microorganism causin BSIs, in South East of Sweden, 2000-2013


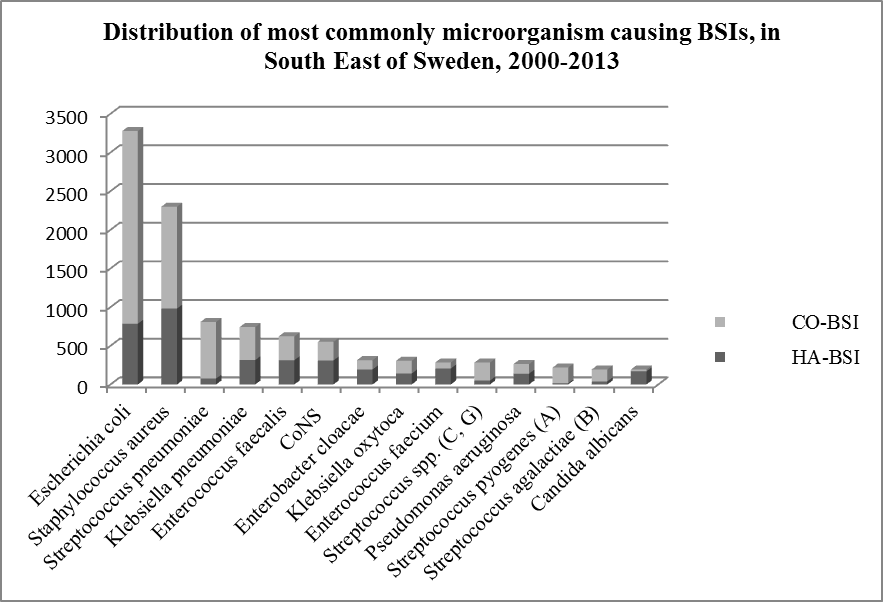


**Data D.** Incidence rate of BSIs (HA and CO) 2000-2013 per 100,000 hospital days

|  |  |  |  |  |  |  |  |  |  |  |  |  |  |  |  |  |  |  |
| --- | --- | --- | --- | --- | --- | --- | --- | --- | --- | --- | --- | --- | --- | --- | --- | --- | --- | --- |
|  |  |  |  |  |  |  |  |  |  |  |  |  |  |  |  | **Linear regression** | | |
|  | **2000** | **2001** | **2002** | **2003** | **2004** | **2005** | **2006** | **2007** | **2008** | **2009** | **2010** | **2011** | **2012** | **2013** | **Change**  **%*** | **Average yearly increase**** | **95% CI** | **P-Value** |
| **BSI** | 166 | 170 | 164 | 178 | 203 | 207 | 218 | 239 | 186 | 269 | 291 | 296 | 340 | 350 | 111% | 19 | 14.5-22.6 | <0.01 |
| **HA** | 63 | 68 | 56 | 67 | 65 | 66 | 67 | 82 | 61 | 87 | 92 | 94 | 101 | 106 | 70% | 4 | 3.0-5.7 | <0.01 |
| **CO** | 103 | 102 | 107 | 111 | 138 | 141 | 151 | 157 | 125 | 181 | 199 | 202 | 239 | 243 | 129% | 14 | 11.2-17.2 | <0.01 |
|  |  |  |  |  |  |  |  |  |  |  |  |  |  |  |  |  |  |  |

* Change in rate from 2000-2013 per 100.000 hospital days

** Increased cases per year per 100,000 hospital days (average annual increase %)

| **Data E.** Antibacterials for systemic use excluding metenamine (J01-J01XX05) dispensed to outpatients 2000-2015 measured as defined daily doses per 1,000 inhabitants and day, or dispensed prescriptions per 1,000 inhabitants and year (Swedish Prescribed Drug Register. Swedish eHealth Authority). | | | | | | | | | | | | | | | | |
| --- | --- | --- | --- | --- | --- | --- | --- | --- | --- | --- | --- | --- | --- | --- | --- | --- |
|  | 2000 | 2001 | 2002 | 2003 | 2004 | 2005 | 2006 | 2007 | 2008 | 2009 | 2010 | 2011 | 2012 | 2013 | 2014 | 2015 |
| Defined daily doses per 1,000 inhabitants and day | 11.6 | 11.3 | 10.9 | 10.9 | 10.5 | 10.8 | 11.3 | 12.0 | 11.6 | 11.3 | 11.5 | 11.3 | 11.1 | 10.8 | 10.5 | 10.3 |
| Dispensed prescriptions per 1,000 inhabitants and year | 421.5 | 410.7 | 391.1 | 385.4 | 364.2 | 367.7 | 379.6 | 396.0 | 371.0 | 357.9 | 359.2 | 349.1 | 339.7 | 322.5 | 318.0 | 313.0 |

| **Data F.** Amount of antibacterials for systemic use (J01) used at hospital wards and policlinics measured in defined daily doses (DDD) per 1,000 hospital day (Swedish Prescribed Drug Register. Swedish eHealth Authority). | | | | | | | | | | | | | | |
| --- | --- | --- | --- | --- | --- | --- | --- | --- | --- | --- | --- | --- | --- | --- |
|  | 2000 | 2001 | 2002 | 2003 | 2004 | 2005 | 2006 | 2007 | 2008 | 2009 | 2010 | 2011 | 2012 | 2013 |
| Tetracyclines (J01A) | 58.5 | 64.0 | 61.3 | 71.8 | 82.8 | 91.3 | 78.0 | 100.2 | 87.6 | 87.1 | 80.3 | 96.2 | 102.0 | 82.6 |
| Penicillins with extended spectrum (J01CA) | 45.0 | 44.1 | 49.3 | 54.4 | 58.5 | 67.2 | 61.7 | 63.1 | 50.3 | 64.2 | 60.2 | 61.3 | 59.3 | 68.6 |
| Beta-lactamase sensitive penicillins (J01CE) | 41.3 | 39.7 | 37.8 | 39.1 | 37.4 | 38.4 | 45.2 | 42.6 | 41.1 | 45.7 | 43.9 | 54.9 | 59.9 | 62.2 |
| Beta-lactamase resistant penicillins (J01CF) | 69.6 | 65.9 | 67.8 | 69.6 | 63.4 | 71.0 | 70.1 | 72.6 | 65.8 | 90.8 | 90.8 | 106.1 | 122.9 | 130.1 |
| Combinations of penicillins (J01CR) | 5.4 | 6.9 | 7.9 | 10.0 | 12.1 | 14.1 | 19.2 | 21.8 | 26.4 | 31.3 | 41.1 | 50.9 | 46.2 | 50.4 |
| Cephalosporins (J01DB-DE) | 81.7 | 76.5 | 52.0 | 75.9 | 88.0 | 89.5 | 92.7 | 95.3 | 81.2 | 93.9 | 90.9 | 89.8 | 94.4 | 98.0 |
| Carbapenems (J01DH) | 20.3 | 18.7 | 18.8 | 24.5 | 24.6 | 25.3 | 27.8 | 30.5 | 31.3 | 36.1 | 42.0 | 46.4 | 47.0 | 48.6 |
| Sulfonamides and trimethoprim J01E) | 10.8 | 12.4 | 13.1 | 13.5 | 16.4 | 17.9 | 22.0 | 27.0 | 25.4 | 22.8 | 20.1 | 18.7 | 17.6 | 15.7 |
| Macrolides. lincosamides and streptogramins (J01F) | 14.6 | 14.7 | 15.6 | 17.8 | 17.3 | 23.8 | 31.5 | 29.7 | 23.5 | 27.2 | 30.5 | 32.6 | 32.7 | 35.3 |
| Aminoglycosides (J01GB) | 4.9 | 3.8 | 3.8 | 5.2 | 5.9 | 5.4 | 5.0 | 8.1 | 6.2 | 6.2 | 9.7 | 11.5 | 11.0 | 9.8 |
| Fluoroquinolones (J01MA) | 50.3 | 53.7 | 61.4 | 67.4 | 62.7 | 68.0 | 70.4 | 65.0 | 56.4 | 62.9 | 66.4 | 70.5 | 63.5 | 63.6 |
| Vancomycin (J01XA01) | 3.9 | 4.3 | 4.0 | 4.4 | 4.6 | 7.2 | 8.6 | 8.0 | 7.0 | 8.4 | 9.9 | 10.3 | 9.6 | 13.0 |
| Other | 18.2 | 18.4 | 19.1 | 17.2 | 16.7 | 20.6 | 20.5 | 21.9 | 21.7 | 22.2 | 23.2 | 25.8 | 26.8 | 31.3 |
| Total amount of antibacterials for systemic use (J01) | 424.4 | 423.2 | 411.8 | 470.6 | 490.3 | 539.8 | 552.8 | 585.8 | 524.0 | 598.9 | 609.3 | 675.1 | 692.9 | 709.2 |

| **Data G.** Amount of antibacterials for systemic use (J01) used at hospital wards and policlinics measured in defined daily doses (DDD) per episode of care. (Swedish Prescribed Drug Register. Swedish eHealth Authority). | | | | | | | | | | | | | | |
| --- | --- | --- | --- | --- | --- | --- | --- | --- | --- | --- | --- | --- | --- | --- |
|  | 2000 | 2001 | 2002 | 2003 | 2004 | 2005 | 2006 | 2007 | 2008 | 2009 | 2010 | 2011 | 2012 | 2013 |
| Tetracyclines (J01A) | 0.33 | 0.35 | 0.34 | 0.37 | 0.43 | 0.45 | 0.37 | 0.49 | 0.48 | 0.39 | 0.37 | 0.43 | 0.45 | 0.37 |
| Penicillins with extended spectrum (J01CA) | 0.26 | 0.24 | 0.27 | 0.28 | 0.30 | 0.33 | 0.29 | 0.31 | 0.27 | 0.29 | 0.28 | 0.27 | 0.26 | 0.30 |
| Beta-lactamase sensitive penicillins (J01CE) | 0.24 | 0.22 | 0.21 | 0.20 | 0.19 | 0.19 | 0.21 | 0.21 | 0.22 | 0.21 | 0.20 | 0.25 | 0.26 | 0.27 |
| Beta-lactamase resistant penicillins (J01CF) | 0.40 | 0.36 | 0.38 | 0.36 | 0.33 | 0.35 | 0.33 | 0.35 | 0.36 | 0.41 | 0.42 | 0.47 | 0.54 | 0.58 |
| Combinations of penicillins (J01CR) | 0.03 | 0.04 | 0.04 | 0.05 | 0.06 | 0.07 | 0.09 | 0.11 | 0.14 | 0.14 | 0.19 | 0.23 | 0.20 | 0.22 |
| Cephalosporins (J01DB-DE) | 0.47 | 0.42 | 0.29 | 0.39 | 0.45 | 0.45 | 0.44 | 0.47 | 0.44 | 0.42 | 0.42 | 0.40 | 0.42 | 0.43 |
| Carbapenems (J01DH) | 0.12 | 0.10 | 0.11 | 0.13 | 0.13 | 0.13 | 0.13 | 0.15 | 0.17 | 0.16 | 0.20 | 0.21 | 0.21 | 0.21 |
| Sulfonamides and trimethoprim J01E) | 0.06 | 0.07 | 0.07 | 0.07 | 0.08 | 0.09 | 0.10 | 0.13 | 0.14 | 0.10 | 0.09 | 0.08 | 0.08 | 0.07 |
| Macrolides, lincosamides and streptogramins (J01F) | 0.08 | 0.08 | 0.09 | 0.09 | 0.09 | 0.12 | 0.15 | 0.15 | 0.13 | 0.12 | 0.14 | 0.15 | 0.14 | 0.16 |
| Aminoglycosides (J01GB) | 0.03 | 0.02 | 0.02 | 0.03 | 0.03 | 0.03 | 0.02 | 0.04 | 0.03 | 0.03 | 0.04 | 0.05 | 0.05 | 0.04 |
| Fluoroquinolones (J01MA) | 0.29 | 0.29 | 0.34 | 0.34 | 0.32 | 0.34 | 0.33 | 0.32 | 0.31 | 0.28 | 0.31 | 0.32 | 0.28 | 0.28 |
| Vancomycin (J01XA01) | 0.02 | 0.02 | 0.02 | 0.02 | 0.02 | 0.04 | 0.04 | 0.04 | 0.04 | 0.04 | 0.05 | 0.05 | 0.04 | 0.06 |
| Other | 0.10 | 0.10 | 0.11 | 0.09 | 0.09 | 0.10 | 0.10 | 0.11 | 0.12 | 0.10 | 0.11 | 0.12 | 0.12 | 0.14 |
| Total amount of antibacterials for systemic use (J01) | 2.42 | 2.31 | 2.29 | 2.41 | 2.52 | 2.68 | 2.60 | 2.86 | 2.86 | 2.69 | 2.83 | 3.02 | 3.05 | 3.14 |

**Data H.** Mortality per 100,000 hospital admission (BSI, HA, CO)

|  |  |  |  |  |  |  |  |  |  |  |  |  |  |  |  | **Linear regression** | | |
| --- | --- | --- | --- | --- | --- | --- | --- | --- | --- | --- | --- | --- | --- | --- | --- | --- | --- | --- |
|  | **2000** | **2001** | **2002** | **2003** | **2004** | **2005** | **2006** | **2007** | **2008** | **2009** | **2010** | **2011** | **2012** | **2013** | **Change**  **%*** | **Average yearly increase **** | **95% CI** | **P-value** |
| **BSI** | 142 | 121 | 106 | 99 | 129 | 146 | 118 | 143 | 133 | 174 | 165 | 175 | 174 | 205 | 44.5 | 8 | 4.2-10.9 | <0.01 |
| **HA** | 79 | 56 | 49 | 40 | 64 | 56 | 55 | 63 | 59 | 72 | 74 | 85 | 68 | 81 | 2.7 | 2 | 0.02-4.2 | 0.05 |
| **CO** | 63 | 65 | 57 | 58 | 65 | 91 | 62 | 80 | 74 | 102 | 91 | 89 | 106 | 124 | 97.1 | 5 | 3.3-7.6 | <0.01 |

* Change in rate from 2000-2013 per 100,000 hospital admission

** Increased cases per year per 100,000 hospital admission, (average annual increase %)

**Data I.** Bloodstream infection - incidence per hospital admission (adjusted for, sex, age and comorbidities Poisson regression).

|  | **HA** |  |  | **CO** |  |  |
| --- | --- | --- | --- | --- | --- | --- |
|  | IRR | 95% CI | P-value | IRR | 95% CI | P-value |
| **2000** | 1 |  |  | 1 |  |  |
| **2001** | 0.97 | 0.85-1.11 | 0,68 | 0.99 | 0.83-1.17 | 0.89 |
| **2002** | 0.93 | 0.82-1.10 | 0,31 | 0.87 | 0.73-1.04 | 0.12 |
| **2003** | 0.88 | 0.77-1.01 | 0,06 | 0.93 | 0.78-1.11 | 0.41 |
| **2004** | 1.06 | 0.93-1.21 | 0,37 | 0.94 | 0.79-1.12 | 0.48 |
| **2005** | 0.95 | 0.83-1.08 | 0,40 | 0.88 | 0.73-1.05 | 0.15 |
| **2006** | 0.97 | 0.85-1.10 | 0,64 | 0.88 | 0.73-1.05 | 0.15 |
| **2007** | 0.94 | 0.83-1.07 | 0,34 | 0.95 | 0.80-1.13 | 0.57 |
| **2008** | 0.74 | 0.65-0.84 | <0,01 | 0.79 | 0.66-0.94 | <0.01 |
| **2009** | 0.79 | 0.69-0.89 | <0,01 | 0.84 | 0.71-1.00 | <0.05 |
| **2010** | 0.86 | 0.76-0.97 | <0,02 | 0.87 | 0.73-1.02 | 0.09 |
| **2011** | 0.80 | 0.70-0.90 | <0,01 | 0.81 | 0.69-0.96 | <0.02 |
| **2012** | 0.82 | 0.73-0.92 | <0,01 | 0.81 | 0.69-0.95 | <0.02 |
| **2013** | 0.83 | 0.74-0.94 | <0,01 | 0.89 | 0.76-1.05 | 0.17 |

**Data J.** Mortality - Incidence per hospital admission (adjusted for, sex, age and comorbidities Poisson regression).

|  | **HA** |  |  | **CO** |  |  |
| --- | --- | --- | --- | --- | --- | --- |
|  | IRR | 95% CI | P-value | IRR | 95% CI | P-value |
| **2000** | 1 |  |  | 1 |  |  |
| **2001** | 0.85 | 0.70-1.04 | 0,11 | 0.98 | 0.79-1.22 | 0.88 |
| **2002** | 0.90 | 0.73-1.10 | 0,29 | 1.03 | 0.84-1.27 | 0.75 |
| **2003** | 0.76 | 0.62-0.94 | 0,01 | 0.85 | 0.69-1.05 | 0.13 |
| **2004** | 0.91 | 0.74-1.11 | 0,35 | 1.01 | 0.82-1.24 | 0.94 |
| **2005** | 0.83 | 0.68-1.02 | 0,07 | 1.12 | 0.93-1.37 | 0.24 |
| **2006** | 0.89 | 0.73-1.09 | 0,25 | 0.81 | 0.66-1.00 | <0.05 |
| **2007** | 0.84 | 0.69-1.03 | 0,09 | 0.97 | 0.79-1.18 | 0.75 |
| **2008** | 0.86 | 0.71-1.05 | 0,14 | 0.91 | 0.75-1.11 | 0.37 |
| **2009** | 0.75 | 0.61-0.91 | <0,01 | 0.85 | 0.70-1.03 | <0.1 |
| **2010** | 0.76 | 0.63-0.93 | <0,01 | 0.86 | 0.71-1.04 | 0.12 |
| **2011** | 0.80 | 0.66-0.96 | <0,02 | 0.85 | 0.70-1.03 | <0.09 |
| **2012** | 0.81 | 0.68-0.98 | <0,03 | 0.91 | 0.76-1.09 | 0.32 |
| **2013** | 0.73 | 0.61-0.89 | <0,01 | 0.89 | 0.74-1.07 | 0.21 |

**Data K.** Comorbidities among blood cultured patients (n=109,983)

|  | **Linear regression** | | | | |
| --- | --- | --- | --- | --- | --- |
| **Numbers of comorbidities** | **Yearly change** | **95% CI** | | **P-value** |  |
| 0 | -40.7 | -50.3--31.2 |  | < 0,00 |  |
| 1 | 85.7 | 57.6-113.7 |  | < 0,00 |  |
| 2 | 73.7 | 59.2-88.2 |  | < 0,00 |  |

| **Data L.** Quality indicators of antibacterials for systemic use (J01) for outpatient use as defined by ESAC (European Surveillance of Antimicrobial Consumption) REF2007. Measured in defined daily doses per 1,000 inhabitants and day; or percentage of total amount of J01 measured in DDD, or as specified fraction measured in DDD. (Swedish Prescribed Drug Register. Swedish eHealth Authority). | | | | | | | | | | | | | | |
| --- | --- | --- | --- | --- | --- | --- | --- | --- | --- | --- | --- | --- | --- | --- |
|  | 2000 | 2001 | 2002 | 2003 | 2004 | 2005 | 2006 | 2007 | 2008 | 2009 | 2010 | 2011 | 2012 | 2013 |
| Antibacterials for systemic use (J01)* | 12.79 | 12.51 | 12.07 | 12.10 | 11.86 | 12.07 | 12.67 | 13.22 | 12.71 | 12.40 | 12.43 | 12.27 | 12.19 | 11.83 |
| Antibacterials for systemic use excluding  metenamine (J01-J01XX05) * | 11.64 | 11.33 | 10.89 | 10.88 | 10.53 | 10.78 | 11.35 | 11.96 | 11.61 | 11.34 | 11.45 | 11.25 | 11.15 | 10.77 |
| Penicillins (J01C)* | 6.78 | 6.60 | 6.29 | 6.11 | 5.74 | 5.80 | 6.10 | 6.60 | 6.55 | 6.40 | 6.24 | 5.99 | 5.97 | 5.85 |
| Cephalosporins (J01D)* | 0.50 | 0.46 | 0.45 | 0.45 | 0.39 | 0.37 | 0.37 | 0.36 | 0.32 | 0.25 | 0.26 | 0.26 | 0.24 | 0.23 |
| Macrolides, lincosamides and streptogramins (J01F)* | 0.51 | 0.54 | 0.50 | 0.48 | 0.50 | 0.57 | 0.60 | 0.64 | 0.60 | 0.59 | 0.61 | 0.60 | 0.61 | 0.59 |
| Quinolones (J01M)* | 0.88 | 0.86 | 0.85 | 0.89 | 0.89 | 0.92 | 0.94 | 0.90 | 0.83 | 0.85 | 0.85 | 0.81 | 0.74 | 0.69 |
| Betalactamase sensitive penicillins (J01CE)^#^ | 35.6% | 34.6% | 33.1% | 31.3% | 29.4% | 28.9% | 29.2% | 31.0% | 30.3% | 30.4% | 29.3% | 28.8% | 27.5% | 26.6% |
| Combination of penicillins, including betalactamase inhibitors (J01CR)^#^ | 1.9% | 1.8% | 2.0% | 1.9% | 1.8% | 2.1% | 2.3% | 2.4% | 2.8% | 2.9% | 3.2% | 3.0% | 3.0% | 3.0% |
| Third and fourth generation cephalosporins (J01DD+DE)^#^ | 0.1% | 0.1% | 0.1% | 0.2% | 0.2% | 0.1% | 0.1% | 0.2% | 0.2% | 0.2% | 0.2% | 0.3% | 0.3% | 0.3% |
| Fluoroquinolones (J01MA)^#^ | 6.9% | 6.9% | 7.1% | 7.4% | 7.5% | 7.6% | 7.5% | 6.8% | 6.6% | 6.8% | 6.8% | 6.6% | 6.1% | 5.9% |
| Ratio of broad spectrum [(J01CR+DC+DD)+(J01F-J01FA01)] to narrow spectrum (J01CE+DB+FA01) penicillins, cephalosporins and macrolides | 12.2% | 12.3% | 13.6% | 14.0% | 14.9% | 15.9% | 16.8% | 16.5% | 18.6% | 19.7% | 21.2% | 21.6% | 23.5% | 25.8% |

* Defined daily doses per 1,000 inhabitants and day

^#^ Percentage of total amount (J01) measured as defined daily doses
